# Processing impact on tocopherols and triglycerides composition of soybean oil and its deodorizer distillate evaluated by high-performance liquid chromatography

**DOI:** 10.3906/kim-2005-10

**Published:** 2020-12-16

**Authors:** Zahid Hussain LAGHARI, Syed Tufail Hussain SHERAZI, Hamide Filiz AYYILDIZ, Mustafa TOPKAFA, Hüseyin KARA, Sarfaraz Ahmed MAHESAR, Sirajuddin .

**Affiliations:** 1 National Centre of Excellence in Analytical Chemistry, University of Sindh, Jamshoro Pakistan; 2 Department of Basic Pharmaceutical Sciences, Faculty of Pharmacy, Selçuk University, Konya Turkey; 3 Department of Chemistry, Faculty of Science, Selçuk University, Konya Turkey; 4 International Center for Chemical and Biological Sciences, University of Karachi, Karachi Pakistan

**Keywords:** Soybean oil, processing, deodorizer distillate, tocopherols, triglycerides

## Abstract

In the present study, high-performance liquid chromatography (HPLC) was used for the separation of tocopherols and triglycerides of processed soybean oil and deodorizer distillate (DD). The results of normal and reversed-phase modes of HPLC revealed that concentrations of tocopherols and triglycerides content were decreased during the neutralization, bleaching, and deodorization processes. The loss of individual tocopherols ranged between 55.16% and 63.25%. During processing, triglycerides containing stearic-oleic-linoleic (SOL) moieties and palmitic-palmitic-linoleic (PPL) fragments showed greater reduction up to 38.14% and 37.69%, respectively. Among tocopherols and triglycerides; γ-tocopherol and oleic-oleic-oleic (OOO) were found to be in greater concentrations 5.53% and 19.78%, respectively in DD as compared to their counterparts. A maximum reduction of tocopherols was observed in the deodorization step. DD was found to be a rich source of bioactive components; therefore, it could be used for many industrial applications including pharmaceutical formulations, cosmetics, and food industries.

## 1. Introduction

Due to the unique fatty acid profile, soybean oil plays an important role in our daily life. Vegetable oils are processed to remove impurities such as odoriferous compounds, gums, glycerol, waxes, pesticides, heavy metals, and some other minor impurities [u1d42]. The quality of the extracted soybean oil depends on the variety as well as the origin of seeds and conditions of industrial processing (degumming/neutralization, bleaching, and deodorization). During processing, various by-products are obtained and the most useful by-product is deodorizer distillate (DD). It is a rich source of natural bioactive components containing squalene, fatty acids, tocopherols, and sterols [3,4] which could be utilized in pharmaceutical, biodiesel, cosmetic, and food industries [5–7].

During the deodorization process, vegetable oils may undergo polymerization at a high temperature, which could lead to producing several polymers such as oxidized triacylglycerols (ox-TAG), diacylglycerols (DAG), oxidized diacylglycerols (ox-DAG), monoacylglycerols (MAG), oxidized monoacylglycerols (ox-MAG), and free fatty acids [8,9]. These oxidized polymeric compounds greatly affect the quality of the oils. The edible oil plays a very important role in providing energy, essential fatty acids, and serve as a carrier of the fat-soluble vitamins to our body [10]. The vegetable oils such as soybean, canola, sunflower, corn, and rapeseed are natural sources of bioactive compounds and antioxidants such as tocopherols and tocotrienols [11,12]. The tocopherols and tocotrienols are naturally present in many vegetable oils in form of different isomers which are collectively known as tocols (α-, β-, γ-, and δ-tocopherols and α-, β-, γ-, and δ-tocotrienols). Each member of tocols has its function and effect on human health [13,14]. Tocols are potent natural antioxidants and prevent the rancidity of oils during storage and thus increase the shelf life of edible oils. Additionally, tocopherols have an important role in the prevention of many types of diseases such as Parkinson’s disease, ataxia, and various types of cancer are due to deficiency of vitamin E. Around 0.1–1% of phytosterols are commonly present in vegetable oils. However, 1–3 g of these phytosterols per day could provide health benefits to humans. Western countries use approximately 250 mg/day phytosterols mainly derived from vegetables, nuts, cereals, etc. [15]. Triglycerides are the main source of energy in our diet and the major function in our body is to act as carriers for fat-soluble vitamins such as vitamins A, D, E, and K. Food chemists are interested in separating and quantifying these valuable bioactive components with suitable methods and authentic analytical techniques. Gas chromatography (GC) and high-performance liquid chromatography (HPLC) are commonly used for the separation of tocopherols and triglycerides [16,17]. There are many studies reporting the separation and quantification of valuable components by using chromatographic techniques, especially capillary GC. Generally, widely used for fatty acid composition, while HPLC is a reliable and sensitive method for the determination of tocopherols [18–20]. Types and concentrations of tocopherols and triglycerides of processed soybean oil as well as DD depend on the quality of the extracted soybean oil, refining technology, and parameters used during different refining processes. To our knowledge, there has been a shortage of data on triglyceride composition of DD since 1984. Therefore, researchers should focus their attention on evaluating triglyceride composition of different vegetable oil DDs for their proper utilization and setting biomarkers for identification of unknown DD. Also, monitoring of triglyceride composition and tocopherol profile is essential from the economy, processing efficiency, and health points of view. The aim of the present study was to investigate the effect of industrial processing on total and individual levels of tocopherols and triglycerides of processed soybean oil and DD of the same soybean oil by the applications of HPLC using normal and reversed-phase approaches.

## 2. Materials and methods

### 2.1. Reagent and sample collection

Analytical and HPLC grade acetone, acetonitrile, tetrahydrofuran, methyl tert-butyl ether, and n-heptane were obtained from Sigma-Aldrich (St. Louis, MO, USA). Standards of tocopherol such as alpha, beta, gamma, and delta (α-T, β-T, γ-T, and δ-T) tocopherols were obtained from E-Merck (Darmstadt, Germany). The TGs standard kits (TRI19-1KT, Supelco®TGs-Kit), (37 component bulk mixes) were purchased from Supelco (Bellefonte, PA, USA). The soybean crude oil, neutralized oil, bleached oil, deodorized oil, and soybean deodorizer distillate (SB-DD) samples were collected from industries of Karachi, Pakistan. All the oil set samples were kept in the refrigerator at 4 °C until further analysis.

### 2.2. Preparation of standard for stock solution

The stock solutions of standard tocopherols were prepared by dissolving 10 mg of the standard of tocopherols in 50 mL of n-hexane for trial analysis. The stock solutions were further diluted into different concentrations to detect the lower and higher concentrations of tocopherols (0.5 to 20 µg/mL). The tocopherols from the processed soybean oils and DD were quantified with a linear calibration method using single and mixed tocopherol standards based on peak areas. The prepared standards were stored at 4 °C in the refrigerator until further analysis.

### 2.3. Tocopherols composition by NP-HPLC

AOCS Official method Ce 5b-89 [21] was used for the separation of tocopherols from processed soybean oil samples. Tocopherols composition was determined by using NP-HPLC (Agilent 1200 series) system fitted with a fluorescence detector (FLD) (Agilent Technologies Inc., Wilmington, DE, USA). Chemstation B.03.02-2008 data processor was used for the separation of tocopherols. In a 2 mL methanol-acetonitrile solvent system (30:70; v/v), 0.04 g of crude, neutralized, bleached, and deodorized oil was dissolved as per the reported procedure. After the centrifugation, 1 mL of supernatant was injected into HPLC. The same procedure was applied to DD samples but the amount of samples was decreased to 0.0025 g due to the presence of a higher concentration of tocopherols in the DD as per the reported procedure [22]. All prepared samples were stored in amber vials and purged with nitrogen to avoid oxidation till analysis. Ten microliters of this mixture was injected into the LiChrospher Si 100-5 column (250 × 4 mm, 5 μm film thickness, Hichrom, England). On trial analysis, different mobile phase was used such as n-hexane and 2-propanol (96:4, v/v), acetonitrile and methanol (50:50, v/v), and methanol-acetonitrile (1:1, v/v), but good separation results were achieved by the mixture of n-heptane, tert-butyl methyl ether (95:5, v/v) at a flow rate of 1 mL/min with isocratic elution. For excitation, the wavelength of FLD was set at 295 nm, in emission mode, the wavelength of FLD was set at 330 nm. Tocopherols peaks were identified by reference to the chromatograms obtained from the standards and the areas under the peak were quantified; the results were reported as mg/kg.

### 2.4. Triglyceride compositions by RP-HPLC

For triglyceride analysis, 1 g of oil was dissolved in 10 mL acetone and filtered by using a 0.45 µm nylon syringe. Twenty microliters of aliquot was injected into an ACE 5 C18 column (250 × 4 mm), 5 mm particle size. An HPLC Agilent 1200 series (Agilent Technologies Inc., Wilmington, DE, USA), Aberdeen, Scotland), a system with a diode array detector (DAD), and Chemstation B.03.02-2008 data processor were used. Isocratic elution system of acetonitrile and acetone (50: 50, v/v) was used at a flow rate of 1.5 mL/min as the mobile phase [17]. The obtained peaks were detected using a DAD detector set at 215 nm. Identification of individual triglyceride peaks was accomplished by comparing their retention times with those of the authentic standards and their retention time. For accurate calculation of triglyceride composition of soybean oil and its DD by HPLC, percentage concentration, i.e. area of the individual triglyceride was divided by the total area and multiplied by 100. The obtained results were multiplied by the respective response coefficient, which is the ratio between the average value obtained from the triplicate analysis of the standard with a known triglyceride composition and the provided composition.

### 2.5. Statistical analysis

Statistical analysis of the data was carried out using Minitab16 USA software. Data were analyzed by analysis of variance (ANOVA) followed by the Tukey test (P ≤ 0.05 *).* The results are reported as mean ± (SD) of three replicates (each replicate corresponds to a different batch of samples).

## 3. Results and discussion

### 3.1. Tocopherol composition of crude and processed soybean oil

**Figure 1:**
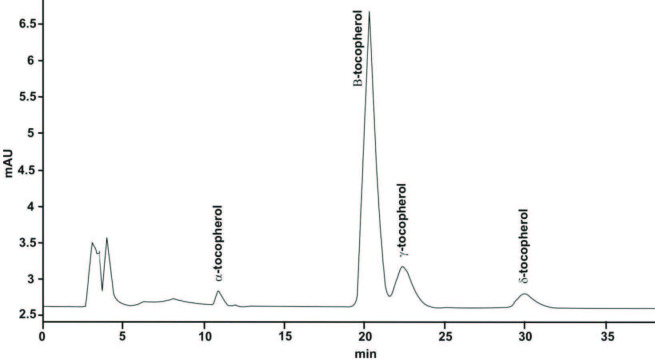
HPLC representative chromatogram of tocopherol composition of deodorized soybean oil.

Table 1 shows the results of α-, β-, γ-, and δ-tocopherols of each processing steps from crude to deodorized soybean oil. Figure 1 shows the representative chromatogram of individual separation of tocopherols of refined soybean oil. According to the NP-HPLC analysis, results show that α- and γ-tocopherols were found in higher concentrations in soybean crude oil and smaller concentration in deodorized oil. In the present study, we have determined four types of tocopherols such as α-, β-, γ-, and δ-tocopherols in each processing samples. In crude oil, the higher concentrations of α-, β-, γ-, and δ-tocopherols were found to be 566.21, 61.70, 657.12, and 120.71 mg/kg, respectively. Ergönül et al. [23] reported nearly the same α-tocopherol level, 557.3 mg/kg, while the quantity of β-, γ-, and δ-tocopherols were slightly lower, 53.0, 599.5, and 118.3 mg/kg, as compared to the present study. In neutralized soybean oil, the concentrations of α-, β-, γ-, and δ-tocopherols were found to be 510.20, 55.12, 598.67, and 108.32 mg/kg, respectively. In comparison to the present study, lower concentrations of α-, β-, γ-, and δ-tocopherols were reported in the neutralization step, 434.3, 38.5, 502.5, and 117.0 mg/kg, respectively. In the bleaching processing step, the concentration of tocopherols was affected by processing conditions. The results of the present study of α-, β-, γ-, and δ-tocopherols were found to be in reduced quantity compared to the neutralization step, 411.79, 40.12, 493.10, and 90.12 mg/kg, respectively. A similar behavior was reported for the neutralization step. In the last step of industrial processing (deodorization), the concentrations of α-, β-, γ-, and δ-tocopherol were reduced and found to be 253.88, 22.67, 255.43, and 50.22 mg/kg, respectively. Ergönül et al. [23] reported higher concentrations of γ- and δ-tocopherol, 434.3 and 74.8 mg/kg, as compared to the present study. In another study, Ayyildiz et al. [24] reported higher concentrations of tocopherols in refined soybean oil for γ- and δ-tocopherols, 540.03 and 277.25 mg/kg, respectively. The different concentrations of tocopherols obtained in this study as compared to the reported studies might be due to different processing conditions and the nature of the seed oil.

**Table 1:** Tocopherol composition of crude and industrially processed soybean oil (mg/kg).

Tocopherols (mg/kg)	Crude oil	Neutralized oil	Bleached oil	Deodorized oil
α- T	566.21 ± 3.01a	510.20 ± 2.02b	411.79 ± 1.10c	253.88 ± 1.02d
β- T	61.70 ± 0.01a	55.12 ± 0.03b	40.12 ± 0.01c	22.67 ± 0.01d
γ- T	657.12 ± 4.01a	598.67 ± 3.02b	493.10 ± 2.03c	255.43 ± 2.02d
δ- T	120.71 ± 0.04a	108.32 ± 1.02b	90.12 ± 0.01c	50.22 ± 0.06d
Total tocopherols	1405.74 ± 3.02b	1272.31 ± 2.02b	1035.13 ± 2.01c	582.2 ± 1.02d

Tocopherol contents indicated with different small letters (a,b,c, and d) are significantly different from each other (P < 0.05)T: Tocopherols

### 3.2. Impact of processing on tocopherols

The industrial processing steps have a great impact on the concentrations of α-, β-, γ-, and δ-tocopherols from (neutralization to deodorization oil) as shown in Table 2. From the crude to neutralization step, a small impact was observed on the individual concentrations of α-, β-, γ- and δ-tocopherols. They were found to be 9.89, 9.81, 8.89, and 10.26%, respectively. From the neutralization to bleaching step, a higher impact on the concentrations of α-, β-, γ­-, and δ-tocopherols were calculated: 19.28, 27.21, 17.63, and 16.80%, respectively. Even a higher impact was calculated from the bleaching to deodorization steps: 38.34, 43.49, 48.19 and 44.27%, on α-, β-, γ­-, and δ-tocopherols, respectively. In the present study, we calculated the overall impact of processing on individual tocopherols from neutralization to deodorization steps. The higher impact (63.25% and 61.2%) was observed on β- and γ-tocopherols and lower impact (55.16% and 58.39%) was observed on α- and δ-tocopherols, respectively.

**Table 2:** Impact on tocopherol composition of crude and industrially processed soybean oil.

Tocopherols	*C-N (%)	*N-B (%)	*B-D (%)	*C-D (%)
α- T	9.89 ± 0.02d	19.28 ± 0.02c	38.34 ± 0.01b	55.16 ± 2.01a
β- T	9.81 ± 0.02d	27.21 ± 1.02c	43.49 ± 1.02b	63.25 ± 3.02a
γ- T	8.89 ± 0.01d	17.63 ± 0.03c	48.19 ± 2.02b	61.21 ± 2.03a
δ- T	10.26 ± 1.02d	16.80 ± 1.02c	44.27 ± 1.04b	58.39 ± 1.02a

Different small letters indicate a significant difference in processing impact on tocopherols samples at P < 0.05. Impact of neutralization (%) = *C-N (crude to neutralized) = Difference/C×100 Impact of bleaching (%) = *N-B (neutralized to bleached) = Difference/N×100 Impact of deodorization (%) = *B-D (bleached to deodorized ) = Difference/B×100 Overall/total Impact (%) = *C-D (crude to deodorized) = Difference/C×100 C: Crude oil, N: Neutralized oil, B: Bleached oil, D: Deodorized oil

### 3.3. Tocopherols composition of deodorizer distillate

**Figure 2:**
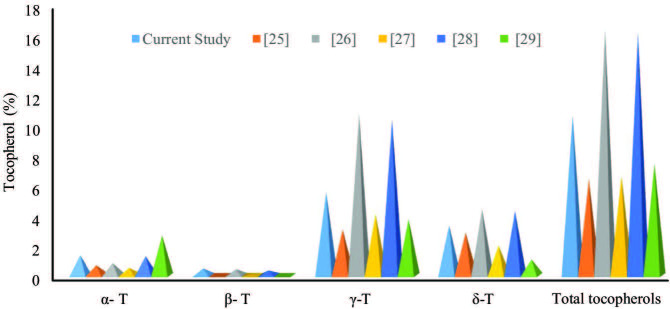
Comparison of tocopherol composition of deodorizer distillate of soybean oil with those of the reported studies.

The results of the tocopherols composition of SB-DD are represented in Figure 2. Results of the present study obtained through HPLC show that γ-tocopherol was found in maximum concentration, while β-tocopherol was found in minimum concentration in SB-DD. The levels of α-, β-, γ-, and δ-tocopherol were found to be 1.31%, 0.44%, 5.53%, and 3.31%, respectively, whereas the concentration of total tocophrols was 10.59%. The results were also compared with those of the reported studies [25–29]. From Figure 2, it is very clear that the concentrations of individual and total tocopheols vary among different studies. The reason for different concentrations of tocopherols reported in different studies may be due to variations in the origin of soybean seed, method of extraction, or processing conditions of soybean oil.

### 3.4. Triglycerides composition of soybean oil

**Figure 3:**
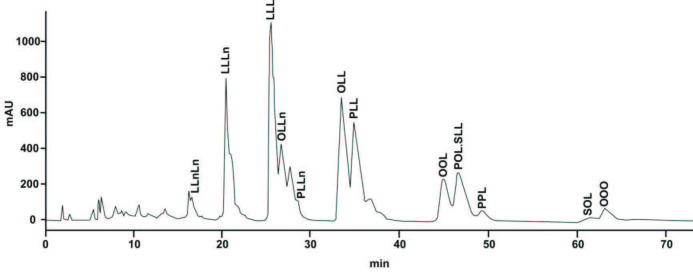
HPLC representative chromatogram of triglyceride composition of deodorized soybean oil.

Triglycerides are commonly obtained from vegetable oils. Triglycerides (oils and fats) are an essential part of the daily diet, a major source of energy, and they act as carriers of fat-soluble vitamins, i.e. A, D, E, and K [17]. In the current study, the triglyceride compositions of crude oil, neutralized oil, bleached oil, and deodorized soybean oil were evaluated. The results of the triglyceride composition of processed soybean oil by RP-HPLC are presented in Table 3 and their chromatogram is shown in Figure 3. In the HPLC chromatogram of the triglycerides of soybean oil and its DD, the Integration function of the Chemstation software provided a quantitative determination. The integration represents the individual peak representative for each triglyceride. Even if the peak is only partially separated from the vertical integration (line drop) from a minimum number of points and by projecting the signal on the baseline, it provides an accurate percentage of individual triglyceride. RP-HPLC analysis revealed twelve different fragments of triglyceride present in processed soybean oil in different concentrations. The triglyceride moieties of soybean oil (crude to deodorized) processed samples were found in an order of linoleic-linolenic-linolenic (LLnLn), linoleic-linoleic-linolenic (LLLn), linoleic-linoleic-linoleic (LLL), oleic-linoleic-linolenic (OLLn), palmitic-linoleic-linolenic (PLLn), oleic-linoleic-linoleic (OLL), palmitic-linoleic-linoleic (PLL), oleic-oleic-linoleic (OOL), palmitic-oleic-linoleic+stearic-linoleic-linoleic (POL+SLL), palmitic-palmitic-linoleic (PPL), stearic-oleic-linoleic (SOL) and oleic-oleic-oleic (OOO). In the current study, the individual levels of triglyceride composition of LLL, OLL, and PLL were present in higher quantities in soybean crude oil 20.36, 16.33, and 13.91%, respectively. While the remaining triglycerides were present in a smaller quantity in soybean crude oil such as LLnLn, LLLn, OLLn, PLLn, OOL, POL + SLL, PPL, SOL, and OOO 3.34, 12.62,7.33, 5.38, 6.51, 9.26, 1.91, 2.91 and 1.11%, respectively. Very little impact was observed in neutralization and bleaching steps on the triglyceride composition of soybean oil. The main triglycerides in neutralized soybean oil, i.e. LLL, OLL, and PLL, were found to be 20.24, 16.20, and 13.71%, respectively. In the bleaching process, LLL, OLL, and PLL were found to be 20.72, 16.58, and 13.94%, respectively. Deodorization is the last processing step of the vegetable oil industry and it was carried out at 180–260 °C. A higher temperature impact on the level of triglyceride concentration was observed in the deodorization step. The loss of concentration of each triglyceride was presented in order of series such as LLnLn, LLLn, LLL, OLLn, PLLn, OLL, PLL, OOL, POL + SLL, PPL, SOL, and OOO was found to be 3.35, 11.28, 21.28, 7.68, 551, 17.55, 13.99, 6.69, 9.49, 1.19, 1.80, and 0.96%, respectively.

**Table 3:** Triglyceride composition of crude and industrially processed soybean oil (%).

Triglyceride (%)	Crude oil	Neutralized oil	Bleached oil	Deodorized oil
LLnLn	3.34 ± 0.01a	3.29 ± 0.02ab	3.17± 0.01c	3.35 ± 0.02a
LLLn	12.62 ± 0.02ab	12.54 ± 0.03ab	12.80 ± 0.02a	11.28 ± 0.05c
LLL	20.36 ± 0.04bc	20.24 ± 0.01c	20.72 ± 0.09ab	21.28 ± 0.04a
OLLn	7.33 ± 0.03b	7.28 ± 0.01bc	7.34 ± 0.05b	7.68 ± 0.01a
PLLn	5.38 ± 0.03ab	5.30 ± 0.02b	5.36 ± 0.03ab	5.51 ± 0.02a
OLL	16.33 ± 0.05bc	16.20 ± 0.05c	16.58 ± 0.01b	17.55 ± 0.05a
PLL	13.91 ± 0.04ab	13.71 ± 0.04b	13.94 ± 0.02ab	13.99 ± 0.06a
OOL	6.51 ± 0.02bc	6.45 ± 0.04c	6.56 ± 0.02ab	6.69 ± 0.09a
POL + SLL	9.26 ± 0.01b	9.16 ± 0.02c	9.36 ± 0.01ab	9.49 ± 0.02a
PPL	1.91 ± 0.02a	1.88 ± 0.11ab	1.23 ± 0.02b	1.19 ± 0.03c
SOL	2.91 ± 0.01a	2.86 ± 0.02ab	1.88 ± 1.43ab	1.80 ± 0.00b
OOO	1.11 ± 0.01a	1.08 ± 0.02ab	1.05 ± 1.24ab	0.96 ± 0.01b

Triglyceride contents indicated with different small letters (a-d) are significantly different from each other (P < 0.05).

### 3.5. Impact of processing on triglyceride composition of soybean oil

The processing impact on triglyceride from crude oil to processed oil is shown in Table 4. The processing impact was observed on the triglyceride’s concentration from the neutralization to deodorization steps. The overall higher impact was observed only on SOL and PPL, which were 38.14 and 37.69%, respectively. In the neutralization step, a minor impact was observed on LLnLn, LLLn, LLL, OLLn, PLLn, OLL, PLL, OOL, POL + SLL, PPL, SOL, and OOO, which was 1.49, 0.63, 0.58, 0.68, 1.48, 0.79, 1.43, 0.92, 1.07, 1.57, 0.01, and 2.70%, respectively. During the bleaching step, the processing impact was increased on the individual levels of triglyceride on LLnLn and SOL, which were 3.64 and 3.26. The remaining series of triglycerides in the processing, minor variation was observed in LLLn, LLL, OLLn, PLLn, OLL, PLL, OOL, POL + SLL, PPL, and OOO. In the deodorization step, the significant impact on triglycerides fragments such as LLnLn, LLLn, LLL, OLLn, PLLn, OLL, PLL, OOL, POL + SLL, PPL, SOL and OOO were found to be 5.67, 11.87, 2.70, 4.63, 2.79, 5.85, 0.35, 1.98, 1.38, 3.25, 4.21, and 8.57%, respectively. Therefore, the loss of nutritive value of refined soybean oil was observed by the influence of the refining conditions and optimal parameters of processing industries.

**Table 4:** Impact on triglyceride composition of crude and industrially processed soybean oil.

Triglyceride	C-N (%)	N-B (%)	B-D (%)	C-D (%)
LLnLn	1.49 ± 0.02c	3.64 ± 0.05b	5.67 ±0.06a	0.31 ± 0.01d
LLLn	0.63 ± 0.01d	2.07 ± 0.02c	11.87 ±0.41a	10.61 ± 0.12b
LLL	0.58 ± 0.00b	2.37 ± 0.01ab	2.70 ± 0.02ab	4.51 ± 0.10a
OLLn	0.68 ± 0.01bc	0.82 ± 0.00b	4.63 ± 0.01a	4.77 ± 0.12a
PLLn	1.48 ± 0.03b	1.13 ± 0.01b	2.79 ± 0.02a	2.41 ± 0.01a
OLL	0.79 ± 0.01d	2.34 ± 0.04c	5.85 ± 0.12b	7.47 ± 0.31a
PLL	1.43 ± 0.03a	1.67 ± 0.01a	0.35 ± 0.00c	0.57 ± 0.01b
OOL	0.92 ± 0.02c	1.70 ± 0.02b	1.98 ± 0.01b	2.76 ± 0.02a
POL + SLL	1.07 ± 0.01b	2.18 ± 0.06a	1.38 ± 0.02b	2.48 ± 0.01a
PPL	1.57 ± 0.04c	2.34 ± 0.04bc	3.25 ± 0.04b	37.69 ± 0.42a
SOL	0.01 ± 0.00c	3.26 ± 0.07bc	4.21 ± 0.05b	38.14 ± 1.01a
OOO	2.70 ± 0.03c	2.77 ± 0.01c	8.57 ± 0.13b	13.51 ± 0.34a

C-N: Crude to neutralized oil, N-B: Neutralized to bleached oil, B-D: Bleached to deodorized oil, C-D: Crude to deodorized oil

### 3.6. Triglyceride composition of deodorizer distillate

The triglycerides composition of SB-DD is shown in Table 5. Triglyceride moieties composition was separated by RP-HPLC such as LLnLn, LLLn, LLL, OLLn, PLLn, OLL, PLL, OOL, POL + SLL, PPL, SOL, and OOO. Among all triglycerides, LLL was found in higher concentration and PLLn was in lower concentration in SB-DD. In the current study, levels of the remaining fragments of triglycerides such as LLnLn, LLLn, LLL, OLLn, PLLn, OLL, PLL, OOL, POL + SLL, PPL, SOL, and OOO in DD were 3.98, 10.63, 11.53, 7.60, 4.60, 1.28, 5.10, 16.38, 7.53, 1.99, 9.60, and 19.78%, respectively.

**Table 5:** Triglyceride composition of deodorizer distillate of soybean oil (%).

Triglyceride	SB-DD(%)
LLnLn	3.98 ± 0.16
LLLn	10.63 ± 0.69
LLL	11.53 ± 0.01
OLLn	7.60 ± 0.03
PLLn	4.60 ± 0.17
OLL	1.28 ± 0.11
PLL	5.10 ± 0.21
OOL	16.38 ± 0.74
POL + SLL	7.53 ± 0.47
PPL	1.99 ± 0.02
SOL	9.60 ± 1.24
OOO	19.78 ± 1.81

SB-DD: Soybean deodorizer distillate

## 4. Conclusion

The impact of industrial processing on the bioactive components of soybean oil and DD was evaluated and explored for industrialists to take proper measures to control the loss of bioactive components during each stage of processing and for plant manufacturing companies to develop such designs that can provide the maximum safety of bioactive components, basic structure, and composition of edible oil. The HPLC results indicated that the overall processing is responsible for the loss of total and individual tocopherols and triglycerides, which means that the nutrition value and stability of soybean oil are compromised during processing. Therefore, there is a strong need to improve the processing so that there is either no loss or minimum loss of these valuable components. However, these useful components are collected in the form of DD, which is the richest source of tocopherols that could be used in the pharmaceuticals and food industries.

## References

[ref1] (2019). Quality evaluation of canola oils and deodorizer distillate during industrial processing. Journal of the Chemical Society of Pakistan.

[ref2] (2014). Chemical characterization of canola and sunflower oil deodorizer distillates. Polish Journal of Food and Nutrition Sciences.

[ref3] (2016). Characterization of palm fatty acid distillate of different oil processing industries of Pakistan. Journal of Oleo Science.

[ref4] (2012). Rapid determination of free fatty acid content in waste deodorizer distillates using SB-ATR-FTIR. Journal of the Association of Official Analytical Chemists International.

[ref5] (2014). Aljaboure A. A Green approach for the production of biodiesel from fatty acids of corn deodorizer distillate. RSC Advances.

[ref6] (2014). Separation of free fatty acids from deodorizer distillates using choline hydrogen carbonate and supercritical carbon dioxide. Separation and Purification Technology.

[ref7] (2016). A single-step isolation of squalene from olive oil deodorizer distillates by using centrifugal partition chromatography. Separation Science and Technology.

[ref8] (2013). Application of FTIR spectroscopy for monitoring the stabilities of selected vegetable oils during thermal oxidation. International Journal of Food Properties.

[ref9] (2009). Distribution of monomeric, dimeric and polymeric products of stigmasterol during thermo‐oxidation. European Journal of Lipid Science and Technology.

[ref10] (2017). Study of physicochemical properties of edible oil and evaluation of frying oil quality by Fourier Transform-Infrared (FT-IR) Spectroscopy. Arabian Journal of Chemistry.

[ref11] (2014). Role of vitamin E as a lipid-soluble peroxyl radical scavenger: in vitro and in vivo evidence. Free Radical Biology and Medicine.

[ref12] (2014). Alpha-tocopherol and gamma-tocopherol concentration in vegetable oils. Food Science and Technology.

[ref13] (2009). Validation of a method for simultaneous determination of tocopherols and tocotrienols in cereals using normal phase HPLC. Journal of Animal and Feed Sciences.

[ref14] (2011). Chemopreventive activity of vitamin E in breast cancer: a focus on γ-and δ-tocopherol. Nutrients.

[ref15] (2002). Spreed enriched with plant sterol ester lowers the blood cholesterol and lipo protein with out affecting vitamins A and E in normal and hypercholesterolmic Japanese men and women. The Journal of Nutrition.

[ref16] (2008). Biological effects of oxidized phytosterols: a review of the current knowledge. Progress in Lipid Research.

[ref17] (2015). Evaluation of the triglyceride composition of pomegranate seed oil by RP- HPLC followed by GC-MS. Journal of the American Oil Chemists’ Society.

[ref18] (2007). Isomerization of conjugated linolenic acids during methylation. Chemistry and Physics of Lipids.

[ref19] (2012). Determination of unsaponifiable constituents of deodorizer distillates by GC-MS. Journal of the American Oil Chemists’ Society.

[ref20] (2019). Effect of refining on bioactive composition and oxidative stability of hazelnut oil. Food Research International.

[ref21] (1998). Official methods and recommended practices of The American Oil Chemists’ Society.

[ref22] (2011). Changes of total tocopherol and tocopherol species during sunflower oil processing. Journal of The American Oil Chemists Society.

[ref23] (2014). Changes in α-, β-, γ-and δ-tocopherol contents of mostly consumed vegetable oils during refining process. CyTA-Journal of Food.

[ref24] (2015). Evaluation of fatty acid composition, tocols profile, and oxidative stability of some fully refined edible oils. International Journal of Food Properties.

[ref25] (2019). Qualitative and quantitative evaluation of tocopherols and phytosterols in soybean oil distillate. Journal of Food Biosciences and Technology.

[ref26] (2016). Vegetable oil deodorizer distillate: a rich source of the natural bioactive components. Journal of Oleo Science.

[ref27] (2019). Li Z. Extraction of tocopherol from soybean oil deodorizer distillate by deep eutectic solvents. Journal of Oleo Science.

[ref28] (2001). chromatographic characterization of vegetable oil deodorization distillate. Journal of Chromatography A.

[ref29] (2003). Von Meien OF. Extraction of tocopherols from the deodorized distillate of soybean oil with liquefied petroleum gas. European Journal of Lipid Science and Technology.

